# Application and interpretation of immunophenotyping data in safety and risk assessment

**DOI:** 10.3389/ftox.2024.1409365

**Published:** 2024-10-04

**Authors:** Victor J. Johnson, Michael I. Luster, Andrew Maier, Corey Boles, Eric W. Miller, Daniel E. Arrieta

**Affiliations:** ^1^ Burleson Research Technologies, Inc., Morrisville, NC, United States; ^2^ Luster Assoc. LLC, Morgantown, WV, United States; ^3^ Stantec ChemRisk, Cincinnati, OH, United States; ^4^ Integral Consulting, Inc., Cincinnati, OH, United States; ^5^ Stantec ChemRisk, Raleigh, NC, United States; ^6^ Insight Exposure and Risk Sciences Group, Raleigh, NC, United States; ^7^ Benchmark Risk Group, Grand Rapids, MI, United States; ^8^ Chevron Phillips Chemical Company LP, The Woodlands, TX, United States

**Keywords:** immunophenotyping, immunotoxicity, safety, risk assessment, human health effects

## Abstract

The use of immunophenotyping during immunotoxicity investigations was first popularized in the 1980 s and has since become more integrated into diagnostic and non-clinical assessments. The data provided from immunophenotyping can serve as an initial source of information to guide decisions for additional, more advanced, immunotoxicity testing as well as for human health safety and risk assessment of drugs and chemicals. However, comprehensive guidance describing applications of immunophenotyping data in immunotoxicity investigations is lacking, particularly among regulatory bodies. Therefore, a critical examination is needed for the appropriate interpretations and potential misinterpretations of such data during the assessment of drug safety and chemical risk. As such, the current uses and implications of immunophenotyping data in human health safety and risk assessments has been evaluated to provide additional context for the application of current methodologies and guidelines. In addition, case studies are presented to highlight the challenges of interpreting immunophenotyping results along with incorporating the findings into immunotoxicity investigations. Based on the analyses of current approaches and methodologies, a decision flow is presented for use of immunophenotyping data during risk informed decision making.

## Introduction

The use of immunophenotyping as a diagnostic tool was first popularized in the early 1980 s when it was discovered that acquired immunodeficiency syndrome (AIDS) was associated with a decrease in cluster of differentiation 4 (CD4^+^) T-cells. Subsequently, immunophenotyping was used to monitor disease progression, as well as efficacy of AIDs therapeutics (Barnett et al., 2008). Currently, it is being used to assist in the diagnosis, classification, and treatment monitoring of hematopoietic neoplasms, such as lymphoma, leukemia, and myeloma (Dworzak et al., 2018; Craig and Foon, 2008; Flores-Montgero et al., 2016), primary immunodeficiency diseases, and recovery in patients following stem cell transplantation (Rawat et al., 2019). Historically, immunophenotyping involved a standardized process by which immune cell populations, specifically lymphocyte, monocyte, and granulocyte subsets, are identified and enumerated using antibodies that recognize unique markers or antigens on the cell surface, in the cytoplasm, or in the nucleus. These antibodies are tagged with fluorochromes, isotopes, or enzymes that then allow the cells to be detected. Typically, combinations of cell markers are used to ensure accurate identification of specific cell types which express similar cell surface proteins. For example, cluster of differentiation (CD) 45 is a common leukocyte surface marker and when combined with CD3, flow cytometry can distinguish T lymphocytes from other leukocytes with a high degree of certainty. Use of this method has been extended to identify many cell types including activated vs. non-activated cells, regulatory cells (*e.g.*, T-reg), dendritic cells, and natural killer (NK) cells. While immunophenotyping is commonly used to monitor cells in a clinical setting, it has also been used in non-clinical settings to help understand cellular processes and chemical toxicity. These applications are enabled due to the use of flow cytometry and its ability to rapidly measure multiple parameters with high accuracy and single-cell resolution. In fact, flow cytometry has helped clarify the processes involved in cell proliferation, differentiation, regulation, and eventual death (apoptosis *versus* necrosis) of specific cell populations. A recent and more novel use of flow cytometry is in quantifying binding of cell surface receptor occupancy assays of biopharmaceuticals to assist in generating pharmacokinetic biomarker data (Liang et al., 2016). The technology and informatics are ever evolving as exemplified by the advent of spectral cytometry making it currently possible to simultaneously interrogate >30 markers and perhaps 100 markers in the next few years (Robinson et al., 2023). Flow cytometry is an integral tool for defining and understanding targets and mechanisms of immunotoxicity, essential data for accurate and comprehensive human health risk assessment.

## Immunophenotyping in immunotoxicology

In immunotoxicology, immunophenotyping aims to determine the impact of biologicals, chemicals, or physical agents on immune cell populations at the single cell level as a direct measure of the agent’s effect. It can detect in specific cell populations that are clinically important but not detectable in routine hematological examination such as decreases in NK cells which can lead to a deficit in tumor surveillance or changes in regulatory cell populations that can lead to the development of autoimmune disease. However, analyses of cell populations does not necessarily address the functional health of the immune cells or system. In fact, functional health is an important aspect of safety and risk assessments; numerous assays have been developed to assess functional health of the immune system (Vos, 1980; Descotes, 2006). Further, recent emphasis on human-relevant *in vitro* and *in silico* testing and prediction methods may assist in interpretation of *in vivo* non-clinical immunophenotype changes. For example, collaborations to develop new approach methodologies (NAMs) for immunomodulators have been described, including those related to single-cell immune profiling (Snapkow et al., 2024). Evaluation of the ability of these functional assays to predict immunosuppression has shown that interrogation of more than one functional endpoint is far more powerful than testing single immune functions in isolation (Luster et al., 1988; Luster et al., 1992). From a toxicological standpoint, flow cytometry has been instrumental in understanding the cellular response to xenobiotics such as processes responsible for cell damage and/or cell death and identification of target cell population(s), as well as effects related to protection mechanisms.

Immunophenotyping data can serve as an initial source of information to guide decisions for additional, more advanced, immunotoxicity testing as well as health safety and risk assessment of drugs and chemicals, therefore, a critical examination of the appropriate interpretations and potential misinterpretations of such data during the assessment process is needed. As such, we have evaluated the current uses of immunophenotyping data in human health safety and risk assessments to provide additional context for applications consistent with current methodologies and guidelines. In addition, the case studies presented highlight the challenges faced when interpreting results, including assessing whether the observed effect is adverse based on directional changes in immunophenotypic cell types with no clear physiological consequence, the lack of sensitivity with immune assays based on chemical exposures, and similar effects observed between studies for certain immune cell types with no clear understanding for the underlying cellular mechanisms to inform a mode of action (MOA) assessment. Further, we discuss the importance of immunophenotyping during risk informed decision making for identifying adverse effect onset doses and setting health-based exposure levels for immunosuppressive effects. The goal of this paper is to identify opportunities and issues with the current approach and provide clarity regarding the value of immunophenotyping as a safety and risk assessment endpoint. In the context of occupational and consumer chemical exposures we will refer to these applications as human health risk assessments.

A comprehensive understanding of immunophenotyping methods, approaches, and techniques is necessary to apply data towards human health risk assessments to ensure accurate interpretation of impacts on the immune system. While immunophenotyping as part of routine immunotoxicology screening can be highly informative, there are also some drawbacks. First, there is a perceived lack of sensitivity for detecting immunotoxicity relative to functional tests. This is in part due to large interspecies and intraspecies variability in cell population numbers and is reflected by large ranges and standard deviations. This biological variability is most apparent in cell populations that occur in low frequency, such as NK cells, multinucleated cells, and monocytes, and can be compounded by technical variability resulting from cell losses due to variability in sample preparation, processing, and analyses. Given the variability among all species, it is important to have an established range of absolute counts for selected immune cell populations. Reference values have been established for humans, examples of which are provided in Table 1. Within the ranges shown, differences occur based upon intrinsic and extrinsic factors including gender, age, and smoking status (Apoil et al., 2017; Maecker et al., 2012). For example, leukocytes, as well as lymphocyte subset count, trend down in humans from birth to 18 years. Thus, without age-specific reference values, cell counts in a neonate with an immunodeficiency may appear similar to a healthy 18-year-old (Marquez et al., 2020). Reference values have also been established for experimental animals. The two most common non-human primates (NHPs) used in biomedical research studies are the cynomolgus (*Macaca fascicularis*) and rhesus (*Macaca mulatta*) macaques. Ranges for these NHPs are shown in Table 2, and, like humans, reflect gender- and age-dependent differences. Reference values are also available for rat and mouse strains commonly used in toxicology studies. Table 2 provides historical control data on immunophenotypes from blood (kindly provided by Charles River Laboratories, Inc., Senneville, QC, Canada) while Table 3 shows historical control data for major immune cell populations from the spleen of popular rodent species used in immunotoxicity testing (kindly provided by Burleson Research Technologies, Inc., Morrisville, NC).

**TABLE 1:** Historical control vales of major immunophenotypes in blood of humans^1^ ^,^ ^2^.

		Total T-cells	Helper T-cells	Cytotoxic T-cells	B-cells	NK cells
Adult^3^	Mean	1.47	0.84	0.41	0.25	0.25
	Range	0.68–2.53	0.39–1.62	0.14–0.84^5^	0.09–0.54	0.07–0.63
Pediatric^4^	Mean	3.8	2.8	1.1	1.30	0.3
	Range	2.4–6.9	1.4–5.1	0.6–2.2	0.7–2.5	0.1–1.0

NK – Natural Killer.

1 Values shown are absolute cell counts. Refer to the source provided for explanation of the immunophenotyping strategies used.

2 Data are not exhaustive and are included to be illustrative of general ranges and patterns of the ranges that occur in aspects of biological variability. For clinicians, additional references such as the National Cancer Institute’s (NCI) Common Terminology Criteria for Adverse Events (CTCAE) Version 5.0 is often referenced (NCI, 2017). See Table 4 in this publication.

3 Data from Apoil et al. (2017). Represents 253 normal healthy individuals from 19 to 67 years of age. Values represent combined genders.

4 Data from Comans-Bitter et al. (1997). Represents 105 infants aged 5–9 months.

**TABLE 2:** Historical control values for major immunophenotypes in experimental animal^1^.

Species		Total T-cells		Helper T-cells		Cytotoxic T-cells		B-cells		NK Cells	
		M	F	M	F	M	F	M	F	M	F
Cynomolgus (Mauritius)^2^	Mean	3.01	2.71	1.54	1.47	1.39	1.15	0.86	0.77	0.85	0.69
	Range	0.83–7.79	0.86–7.56	0.14–3.66	0.38–4.15	0.29–4.03	0.27–3.22	0.14–4.14	0.17–4.06	0.05–3.08	0.06–2.78
	n^5^	734	788	719	776	712	778	762	825	728	839
Rhesus^2^	Mean	2.57	1.42	1.42	0.80	1.21	0.62	0.93	0.97	0.70	0.31
	Range	0.89–4.56	0.90–2.63	0.56–2.55	0.53–1.46	0.33–2.41	0.27–1.12	0.41–1.70	0.48–1.56	0.28–1.39	0.14–0.57
	n^5^	18	18	18	18	18	18	18	18	18	18
SD Rat^3^	Mean	4.76	3.77	3.21	2.62	1.87	1.40	3.71	2.20	0.29	0.22
	Range	2.26–9.32	1.08–7.23	1.27–6.55	0.80–5.85	0.88–3.81	0.29–2.59	1.55–9.09	0.77–4.84	0.07–0.80	0.04–0.58
	n^5^	48	54	48	54	48	54	48	54	48	54
Wistar Rat^3^	Mean	3.71	3.13	2.67	2.31	1.24	0.99	2.51	1.52	0.33	0.23
	Range	0.46–7.18	0.44–8.42	0.41–4.85	0.29–5.48	0.17–3.09	0.15–3.02	0.31–6.35	0.15–4.83	0.08–0.97	0.07–0.79
	n^5^	65	66	65	65	65	65	65	66	65	66
CD-1 Mouse^4^	Mean	0.47	0.83	0.34	0.61	0.12	0.21	0.87	1.21	0.07	0.08
	Range	0.05–1.11	0.10–1.85	0.03–0.80	0.08–1.32	0.01–0.29	0.03–0.51	0.04–2.35	0.11–5.70	0.01–0.16	0.00–0.16
	n^5^	32	32	32	32	32	32	32	32	32	32

^1^Values shown are absoute cell counts. Refer to the source provided for explanation of the immunophenotyping strategies used. (xl06/ml): median and.

^2^Historical control daa provided by Charles River Laboratories, Inc. (Senneville, QC, Canada). Cells were gated on the CD45 populations resulting in T-cells (CD45+/CD3+), T-helper cells (CD45+/CD3+/CD4+), T-cytotoxic cells (CD45+/CD3+/CD8+), B-cells (CD45+/CD3-/CD20+), and NK, cells (CD45+/CD3-/CD16+).

^3^Historical control daa provided by Charles River Laboratories, Inc. (Senneville, QC, Canada). Cells were gated on the CD45 populations resulting in T-cells (CD45+/CD3+), T-helper cells (CD45+/CD3+/CD4+), T-cytotoxic cells (CD45+/CD3+/CD8a+), B-cells (CD45+/CD3-/CD45RA+), and NK, cells (CD45+/CD3-/CD161a+).

^4^Historical control daa provided by Charles River Laboratories, Inc. (Senneville, QC, Canada). Cells were gated on the CD45 populations resulting in T-cells (CD45+/CD3+), T-helper cells (CD45+/CD3+/CD4+), T-cytotoxic cells (CD45+/CD3+/CD8+), B-cells (CD45+/CD3-/CD19+), and NK, cells (CD45+/CD3-/NK1.1+).

^5^n = number of animalsexamined.

M = male, F = female.;NK, Natural Killer; SD, Sprague Dawley.

**TABLE 3:** Historical control values for lymphocyte subsets in spleens from rodents commonly used in immunotoxicology^1^.

Species		Immune Cell Population (x10^6^ splenocytes)									
		Total T-cells		Helper T-cells		Cytotoxic T-cells		B-cells		NK cells	
		M	F	M	F	M	F	M	F	M	F
SD Rat^2^	Mean	66.0	63.3	35.0	36.1	25.0	24.4	93.2	83.4	15.8	11.1
	Range	16.9–124.7	12.2–344.9	9.1–65.4	6.5–197.5	7.0–57.4	5.3–130.2	33.9–171.8	17.8–268.8	1.7–81.5	1.0–59.2
	n^4^	50	86	51	87	52	88	53	89	54	90
B6C3F1/N Mouse^3^	Mean		24.8		14.9		8.5		24.7		1.8
	Range		5.27–58.03		3.29–33.6		1.7–19.9		4.84–61.31		0.19–7.51
	n^4^		142		141		141		142		142

^1^Values shown are abslute cell counts. Refer to the source provided for explanation of the immunophenotyping strategies used.

^2^Historical Control daa provided by Burleson Research Technologies, Inc. (Morrisville, NC, United States of America). Cells were gated on the CD45 populations resulting in T-cells (CD45+/CD3+), T-helper cells (CD45+/CD3+/CD4+), T-cytotoxic cells (CD45+/CD3+/CD8a+), B-cells (CD45+/CD3-/CD45RA+), and NK, cells (CD45+/CD3-/CD161a+).

^3^Historical Control daa provided by Burleson Research Technologies, Inc. (Morrisville, NC, United States of America). Cells were gated on the CD45 populations resulting in T-cells (CD45+/CD3+), T-helper cells (CD45+/CD3+/CD4+), T-cytotoxic cells (CD45+/CD3+/CD8+), B-cells (CD45+/CD3-/CD19+), and NK, cells (CD45+/CD3-/NK1.1+).

^4^n = number of animalsexamined.

M = male, F = female; K, Natural Killer; SD, Sprague Dawley.

While the data provided in Tables 1, 2 are presented as absolute cell counts for each population of interest, it is important to consider relative population counts as those data can be used to interpret shifts in subpopulations of immune cells induced by toxic exposures. For example, shifts in the relative representation of T-cell subsets can be used to interpret which subpopulation is targeted by the toxin and responsible for a change in total T-cells. Even if there is no change in total T-cells, there may be shifts in the relative representation of subtypes which would be important for immune defenses. Since the relative counts are derived from absolute counts, only absolute counts are presented in the present work.

## Immunophenotyping methods and approaches

**FIGURE 1:**
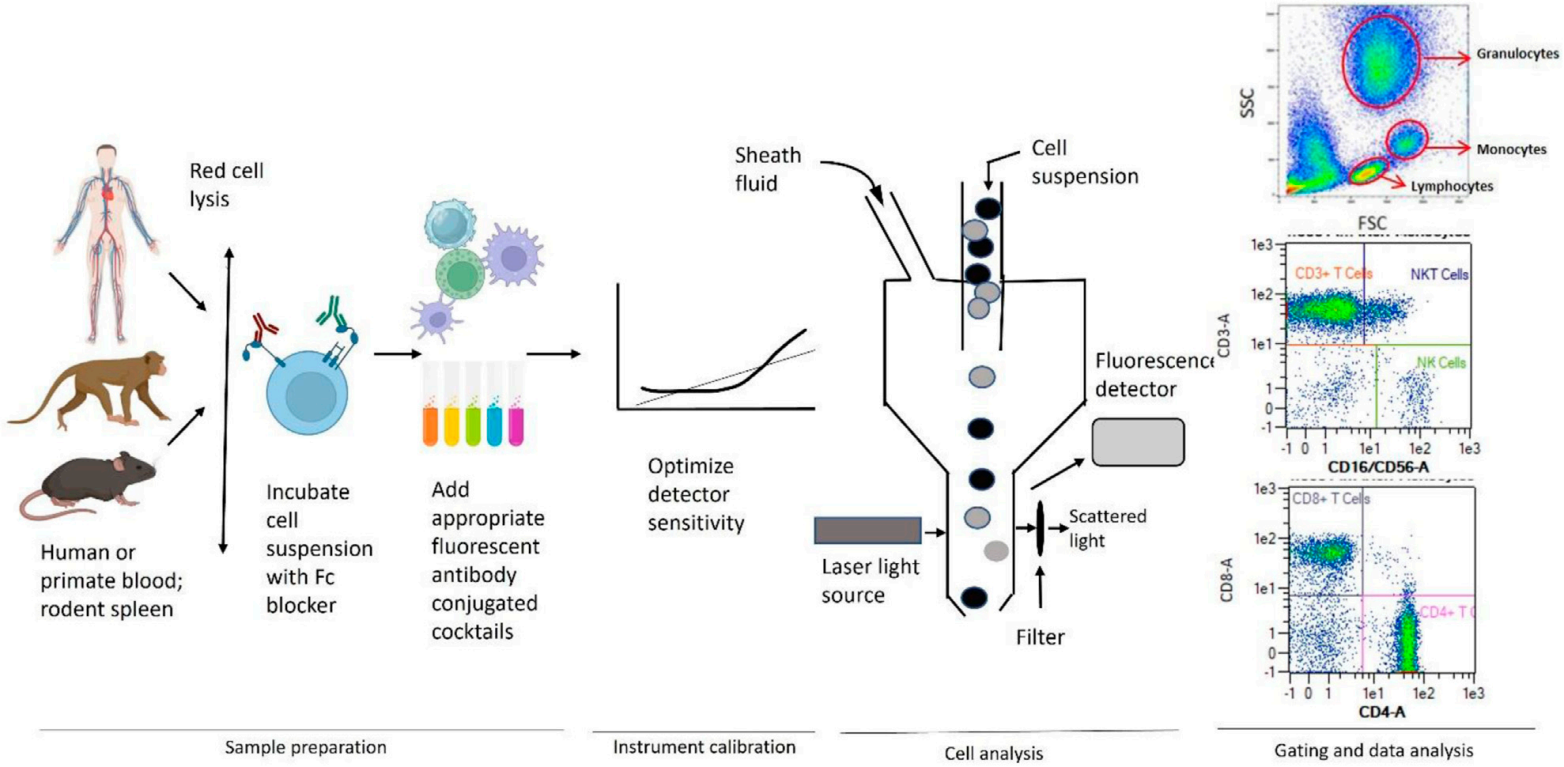
General scheme used for immunophenotyping illustrating sample preparation, instrument calibration, cell analysis, gating, and data analysis by flow cytometry. Adapted from Maecker et al. (2012).

Immunophenotyping studies for immunotoxicity evaluation are usually conducted as an adjunct or follow-up evaluation after white blood cell (WBC) analysis and immune function testing as it provides detailed information on immune cell populations and a better understanding of the mode of action in the presence of functional changes (Wang and Lebrec, 2017). The major steps in a typical experiment using whole blood from human using flow cytometry are shown in Figure 1. Information on immune cell population density and distribution (*in vivo* or *in vitro* exposures) as well as direct cytotoxicity (*in vitro* exposures) are obtained.

Peripheral whole blood is used in studies in humans or non-human primates while whole blood and/or single cell preparations from the spleen or, in some instances, the thymus and/or lymph nodes, are used in laboratory rodents and some larger mammals (*e.g.,* dogs). This technique involves labeling cells with monoclonal antibodies covalently bound to fluorochromes with disparate excitation and/or emission wavelengths that allow for spectral separation and classification of numerous cell types simultaneously. These monoclonal antibodies typically recognize surface antigens (*i.e.,* CD molecules) that are expressed by cells of the immune system. It is rare for a CD marker to be exclusively expressed on a single immune cell type; therefore, it is necessary to use a combination of CD-specific antibodies to discriminate at the single cell level. The more complex the panel of antibodies, the finer the definition of the population being interrogated. Using a flow cytometer allows for the excitation and detection of the fluorochromes at multiple wave lengths simultaneously, thereby providing a rapid and effective method of quantitating numerous cell types (*i.e.,* multicolor analysis) in a single sample. Substantial effort has been devoted to designing multiplex immunophenotyping approaches for human whole blood. One such approach is available commercially that capitalizes on a single 8-color cocktail of antibodies to discriminate leukocytes in human whole blood into 11 distinct immune cell populations including total T-cells, T-helper cells, T-cytotoxic cells, natural killer T-cells (NKT), B-cells, NK cells, eosinophils, neutrophils, and monocytes (classical, intermediate, and non-classical). This strategy provides a valuable tool for determining the effects of environmental and occupational exposures, as well as *in vitro* treatments on human immune cell populations. A commonly used strategy in rodent studies is to gate the mononuclear cell population on the CD45^+^ leukocytes, which are then used to establish subsets of T-cells, B-cells, NK cells, T-helper cells, and T-cytotoxic cells using progressive gating.

Given the extensive array of sources for flow cytometers and associated reagents (*e.g.,* antibodies, staining buffers, fixation buffers) and the fact that laboratories can use commercially or in-house prepared regents, there exists the potential for multiple sources of technical variability within and between laboratories, in addition to the high degree of skilled labor required. As such, the primary areas where variability may occur when applying this technique are reagents, sample handling, instrument setup, and data analysis, including gating strategies. The effects of changes in these variables can be significant between laboratories although the standardization and control of staining reagents using preconfigured lyophilized-reagent plates or antibody cocktails, and standardized data analysis will help to decrease intra- and interlaboratory variability. It should be noted that currently there is a paucity of detail in regulatory guidance documents for the selection of immunophenotyping strategies, methods of data collection, and interpretation of findings. Therefore, effective utilization of immunophenotyping data in human health safety and risk assessment should be delegated to subject matter experts with a thorough understanding of the impacts of study design, cell isolation and staining strategies, flow cytometer instrumentation, and gating strategies as well as how cell population changes relate to immunotoxicity.

## Safety and risk assessment considerations

Safety and risk assessment processes can vary across regulatory applications, with guidance available from most agencies. Such assessments share a foundational set of steps from the toxicology perspective that include hazard characterization, dose-response assessment, and risk characterization (NRC, 2009). The hazard characterization step includes a process of critical review of the health effects literature including epidemiology, toxicology, mechanistic, and other studies. A defined process for data identification, evaluation, and integrations is typically applied using approaches such as those embedded in systematic review methodologies (NTP, 2019; EPA, 2021). This process yields a decision as to whether there is an effect of potential concern (*e.g.,* immunotoxicity) for relevant use scenarios.

The determination of hazard potential is a qualitative step that supports a second step; the development of a quantitative dose benchmark for safety or risk assessment [*i.e.,* a health-based exposure limit (HBEL)]. The derivation of an HBEL relies on analysis of the dose-response behavior for the adverse effect of interest. This process yields a point of departure (POD) that is the starting point for extrapolation to a dose below which lies the estimated onset dose for the adverse effect in susceptible portions of the population. In most cases, developing HBELs requires extrapolation from the POD to an estimated population no effect dose by dividing the POD value by adjustment factors that account for biological variability and data uncertainties. The HBEL can then be used as a comparator to exposure for scenarios under review to determine if exposure has exceeded the HBEL and thus has increasing potential to pose a safety concern or health risk. This comparison of the HBEL and exposure is typically reflected in the risk characterization step of the process.

Data on blood cell counts and immunophenotyping are often collected in clinical or non-clinical studies for new drugs and chemicals requiring regulatory approval. However, for a variety of reasons relatively unique to immunophenotyping, the significance of these studies within the basic risk assessment paradigm are open to interpretation. One key challenge reflects considerations for defining a degree of effect that would be considered as the onset of an adverse health effect. This consideration reflects the selection of the risk assessment critical effect (most sensitive adverse effect or its immediate precursor) and POD. An integrated framework for identifying safe dose levels for first-in-human (FIH) trials of potential immunomodulatory therapeutics for clinical applications has been recently published (Matsumoto et al., 2024). The proposed decision tree for starting dose selection incorporates a tiered safety/risk assessment based on 1) mode of action and the related clinical experience, 2) relevancy of *in vitro* human assays and/or *in vivo* animal studies, and 3) clinical safety risk based on nonclinical toxicology studies, thus allowing for immunomodulatory biologics to diverge from the standard approach based on the minimum anticipated biological effect level (MABEL) to other approaches, including one based on a non-clinical no observed adverse effect levels (NOAEL).

To our knowledge there are no specific quantitative cut points for any immune cell population change or specific immunophenotyping finding that have a consensus definition as an “adverse” effect for the purposes of population level risk assessments. One example which has come closest to providing a quantitative cut point is provided by data on CD4^+^ T cells counts and human immunodeficiency virus (HIV) infection. Normal CD4 counts for healthy adults and teens are approximately 500 to 1,200 cells per cubic millimeter (mm^3^) with low CD4 count considered <500 cells/mm^3^. In cases of patients with HIV, a low CD4 count means that HIV has weakened the immune system. A CD4 count of ≤ 200 cells/mm^3^ indicates the patient has AIDS and is likely to develop life-threatening infections or cancers. In some cases, low CD4 counts are due to cancer therapy, therapeutics, or unknown etiology and while the risk of developing infections or cancer still exists, the frequency is less. It should be noted that for determination of treatment emergent adverse events (TEAE) in clinical studies for pharmaceuticals there are general guidelines for hematological endpoints and severity grade. For example, the National Cancer Institute’s (NCI) Common Terminology Criteria for Adverse Events (CTCAE) Version 5.0 is often referenced (NCI, 2017). Hematological endpoints based on analysis via immunophenotyping along with the endpoints corresponding grades (*i.e.,* severity of AE) and signs/symptoms reported by NCI have been summarized in Table 4.

**TABLE 4:** Hematological endpoints with corresponding grades as reported by NCI CTCAE. Table has been adapted from CTCAE v5.0 (NCI, 2017)^1^.

Blood element^2^	Grade^3^				
	1	2	3	4	5
CD4 Lymphocyte Decrease	<LLN - 500/µL	<500–200/µL	<200–50/µL	<50/µL	—
Febrile Neutropenia	—	—	ANC <1,000/µL with a single temperature of >38.3°C (101°F) or a sustained temperature of ≥38°C (100.4°F) for >1 h	Life-threatening consequences; urgent intervention indicated	Death
Eosinophil Count Increase	>ULN and >Baseline	—	Steroids initiated	—	—
Lymphocyte Count Decrease	< LLN - 800/µL	<800–500/µL	<500–200/µL	<200/µL	—
Lymphocyte Count Increase	—	>4,000–20,000/µL	>20,000/µL	—	—
Neutrophil Count Decrease	< LLN - 1,500/µL	<1,500–1,000/µL	<1,000–500/µL	<500/µL	—
White Blood Cell Decrease	<LLN - 3,000/µL	<3,000–2000/µL	<2000–1,000/µL	<1,000/µL	—
White Blood Cell Increase	—	—	>100,000/µL	Clinical manifestations of leukocytosis; urgent intervention indicated	Death

1 All units have been converted to cells per microliter (cells/µL).

2 Finding based on laboratory test results of blood specimen, except for febrile neutropenia which is also defined by body temperature.

3 Grade refers to the severity of the AE, of which the CTCAE list Grades 1 through 5 with unique clinical descriptions of severity for each AE based on a general guideline (NCI, 2017).

ANC = absolute neutrophilic count; LLN = Lower Limit of Normal; ULN = Upper Limit of Normal.

Thus, the interpretation of the adversity of a finding falls back to general principles of safety and risk assessment that reflect the goal of protecting against a clinical or morphological change that impairs physiological function or the ability of the biological system to withstand additional challenges to normal function. As such, three pragmatic options for the safety or risk assessor to consider in defining adversity of immunophenotyping data include:

1) Reliance on deviations from the population or individual background range. This approach is challenging because there are substantial interspecies and interindividual variability in immunophenotypes (see Tables 1–3). Thus, while statistical changes in certain cell subpopulations may be adverse, a clear distinction for adversity is difficult to identify. One methodology that has been used is to set a pre-determined benchmark response rate based on the control population variability in applying dose-response to continuous data. In addition, an overall effect on immune function might reflect impacts of a combination of cell type changes with only some having small to moderate individual subtype decrements. Thus, complex patterns of changes may be observed and difficult to interpret. Another complication reflects differences in comparative physiology, that is, reliance on non-clinical data is often required in regulatory testing schemes and consideration of the differences in impact of functional immune system performance across species is uncertain from a comparative biology perspective. This question reflects whether a given percentage change has the same functional effect in humans as a similar change in laboratory animals used in toxicity testing studies. As this is largely a question of interspecies differences in toxicodynamics, an adverse outcome pathways (AOP) perspective that reflects on key event relationships is useful. The framework of demonstrating the operative mode of action (MOA) and AOP then allows for increased use of the International Programme on Chemical Safety (IPCS) Chemical Specific Adjustment Factor (AF) methodology to replace default AFs for interspecies differences with data driven effective dose ratios (IPCS, 2005).
2) Another option is to rely on existing guidelines and risk assessment precedent. Several guidance documents are available for assessing immunotoxicity (Table 5). However, these guidelines provide general descriptions for consideration of immunophenotyping and are not definitive nor do they provide quantitative adverse effect cut-off values. Although there may be individual chemical risk assessments that have relied on immunophenotyping data for selecting a POD, we did not find any examples of immunophenotyping data, in contrast to hematological data, serving as the final basis of HBELs among current U.S. Environmental Protection Agency (EPA) reference doses. Thus, additional effort to define regulatory precedent of pre-defined adversity determination is needed.
3) Lastly, the examination of clinical relevance of changes from a pathophysiology perspective can be performed. This seems the most viable approach given the challenges noted for options 1 and 2 above. This approach not only has the strongest opportunity for evidence integration, but also requires support from studies on changes in the incidence of immune-mediated disease including autoimmunity, immune-mediated asthma, infectious diseases, or certain types of cancers, relative to quantifiable change in immune cell populations. In many cases, a MOA analysis approach linked to an AOP is ideal. The identification of early key events and linkage to the adverse biology can be examined using approaches derived from the modified Bradford Hill considerations as applied to chemical risk assessment (Meek et al., 2014). Consultation with an experienced immunologist and/or toxicologist with specific experience evaluating toxicity to the immune system is typically required to support such judgements. The level of confidence in developing an adversity decision is based on the degree of change, as well as the pattern of subtypes affected. The case studies presented in the last section highlight the type of evidence integration approach that has been applied in such complex decisions regarding adversity determinations.

**TABLE 5:** Regulatory and published scientific guidance documents describing the incorporation of immunophenotyping data into immunotoxicity investigations.

Agency, organization, or author	Relevant guidance or method (Year published)	Specific immunophenotyping endpoints	Data interpretation guidance
ATSDR	Guidance for the Preparation of Toxicological Profiles (ATSDR, 2018)	Altered T-cell and macrophage activity and leukopenia (leukocyte count)	Minimal - a list of serious and less serious LOAELs for immunological effect endpoints. Specific numeric cutpoints not provided
OECD	Test No. 443: Extended One-Generation Reproductive Toxicity Study (OECD, 2018)^1^	Splenic lymphocyte subpopulation analysis- CD4^+^ and CD8^+^ T-cells, and B-cells^2^	Minimal - due to application only towards Cohort 1A. Guidance only refers to a “shift in the immunological steady state distribution” of CD4^+^, CD8^+^, or thymus derived lymphocytes
EPA	Health Effects Test Guidelines OPPTS 870.7800 Immunotoxicity (EPA, 1998)	Splenic or peripheral-blood analysis- CD4^+^ and CD8^+^ T-cell, and B-cell enumeration	Minimal - results should be evaluated in conjunction with other toxic effects. According to the EP, routine toxicity testing (*e.g.,* histology, organ weights, hematology) alone is not sufficient to predict immunotoxicity alone
	Biochemicals Test Guidelines: OPPTS 880.3550 Immunotoxicity (EPA, 1996)	Total and differential leukocyte count	Minimal - “dysfunction” and “impairment” referenced but not defined. If dysfunction or impairment noted then Tier II immunotoxicity studies required (OPPTS 880:3,800), which included enumeration of T-cell and B-cell lymphocytes subpopulation and macrophage activity
FDA	Guidance for Industry and FDA Reviewers: Immunotoxicity Testing Guidance (FDA, 1999)	Histopathology and humoral response cell surface markers, T-cells (helper and cytotoxic), NK cells, and Macrophages	Minimal - guidance states that “functional assays are generally more important than tests for soluble mediators or phenotyping.” Flow chart for immunotoxicity testing of medical devices is provided
	Guidance for Industry: Nonclinical Evaluation of the Immunotoxic Potential of Pharmaceuticals (FDA, 2023)	NK cells, T-cells, APCs, and other non-specified immune cells	The FDA highlights the importance of aligning with ICH S8 in applying a WoE approach. FDA notes that immune cell populations and subpopulations should be considered when assessing immunosuppression and carcinogenicity or opportunistic infections, immunostimulation, and developmental immunotoxicity
ICH	S8 Immunotoxicity Studies for Human Pharmaceuticals (ICH, 2005)^3^	Identification and/or enumeration of leukocyte subsets and antigen-specific immune responses of lymphocytes	Minimal - allows for the identification of specific cell populations affected and might provide useful clinical biomarkers. A WoE approach is preferred with multiple assessment approaches
NTP	Explanation of Levels of Evidence for Immune System Toxicity (NTP, 2009)	Alterations in cell counts (nothing specified)	Functional effects, as defined as an alteration in the ability of the immune system to respond to a challenge or stimulus, should usually be weighed more heavily than observational parameters such as alterations in cell counts
WHO/IPCS	Guidance for Immunotoxicity Risk Assessment for Chemicals (IPCS, 2012)	Relative or absolute counts of leukocytes, lymphocytes, eosinophils, or neutrophils, including D4+ and CD8^+^ T-cells	Immunophenotyping data is treated as observational data and the assessor should use WoE when relying on the data as singular source When a large number of immunophenotypic markers is examined, an abnormal value in one or two immunophenotypes is likely to result simply from a type 1 error. A more reliable indicator of immunotoxicity would be multiple changes consistent with a specific pattern
Germolec et al. (2022)	Consensus on the Key Characteristics of Immunotoxic Agents as a Basis for Hazard Identification (2022)	Enumeration of B-cells, CD4^+^ and CD8^+^ T-cells, NK cells, and other subpopulations of leukocytes	Key characteristics of immunotoxicants across varying exposure types can be assessed, in part, using immunophenotyping techniques. In addition, immunophenotyping, in combination with other techniques, can be used to assess immunosuppression and alterations in immune cell trafficking

^1^OECD, Test No. 443 was included due to the additional detail described related immunotoxicological assessments, particularly immunophenotyping. Several other OECD, Test No. describe immunotoxicological assessment, but not in as much detail.

^2^For the investigation of pre- an postnatally induced immunotoxic effects among Cohort 1A. Cohort 3 developmental immunotoxicity assessment relies on TDAR.

^3^Several medical and drug regulatry agencies have adopted ICH S8, including the ANVISA (Brazil), COFEPRIS (Mexico), EC (Europe), FDA (US), Health Canada (Canada), MFDS (Republic of Korea), MHLW/PMDA (Japan), MHRA (United Kingdom), and Swissmedic (Switzerland).

Data and assumptions regarding biological variability play an important role in HBEL derivation. In the risk assessment process, POD selection, and ultimately the HBEL derived from it, need to reflect considerations of biological variability and uncertainty in the data. Selecting adverse effect cut points (*e.g.,* POD estimates) and HBEL derivation is particularly challenging for effects that have a high degree of underlying biological variability. This consideration relates to the interplay between selecting a POD and the size of the AFs applied for extrapolation to a population no effect dose. Since the goal is to identify a subthreshold dose for the sensitive portion of the population (*i.e.,* population upper bound NOAELs), the more precise the toxicology data are for the estimate of onset dose the lower the related AFs for extrapolation. For example, data connecting immunophenotyping to immune function gives greater confidence in the POD selection and can reduce the AF for database insufficiency and extrapolation from an adverse effect. In addition, data on biological variability can assist in increasing confidence in determining if a NOAEL in an observed study is close to the human population upper bound NOAEL.

The potential for overestimation of effects is particularly important for findings relevant to WBC subtypes that have low absolute count or relative percentage values. This relates to the consideration that an observed large drop in percentile across dose groups may have limited impact in terms of absolute number of cells. Interpretation of such findings is most problematic for cell types with unclear direct physiological consequences for a given directional change in count (see eosinophil example in the case studies). Another challenging scenario is interpretation of findings when a change in the count of one cell type could be offset by changes in other subtypes with overlapping physiological roles in immune surveillance. One solution to such challenges is knowledge of the MOA specific to a given cell subtype change or observed pattern. Thus, the interplay of expected patterns of subtype changes and a chemical’s MOA can help to make distinctions between an incidental immunophenotype change and one that is biologically meaningful.

It is also challenging to assess the relevance of changes in endpoints that have large endogenous biological variability. One option for assessing such endpoints is a defined degree of departure from the “normal or typical range”. In such cases, a specific percent change from the range can result in a large absolute change in highly variable cell types compared to cell types with a smaller endogenous range. Thus, the impact of a given departure as a percentage from a defined variation from normal *(e.g.,* +/−5% in a certain subtype) may be large for some cell types depending upon where the individual falls in the range at baseline conditions. Further, there is uncertainty defining the cutoff for what ratio or percentage of departure is significant or biologically relevant. Thus, there is a challenge in applying the “departure for the normal range” approach to individuals or small populations, Moreover, there can be substantial differences in functional reserve capacity such that a change from normal may have little functional consequence. The interpretation of adversity associated with departures from a normal range has been established for several clinical testing endpoints in chemical risk assessment (*e.g.,* plasma levels of liver enzymes; degrees of change in red blood cell cholinesterase activity) commonly used for HBEL derivation but not for immunophenotyping data.

## Existing guidance for interpretation of immunophenotyping data

While progression of immunophenotyping methodology for clinical purposes has been somewhat optimized and refined overtime, there remains a general lack of standardized guidance for the use and interpretation of the associated data during safety and risk assessments applied outside of clinical and pharmaceutical investigations (*i.e.,* chemical exposure), especially with the rapid evolution of flow cytometry technology and antibody panels. In addition, because of the large variability in historical control values among human studies, case values may be significantly different from control values while still falling within historically normal ranges, making interpretation of the results difficult further complicating their application to risk assessment (WHO 2012).

Several agencies and organizations have established guidance pertaining to the appropriate study design and data collection for immunotoxicity assessments. These include the U.S. Agency for Toxic Substances and Disease Registry (ATSDR), Organisation for Economic Co-operation and Development (OECD), U.S. EPA, U.S. Food and Drug Administration (FDA), International Council for Harmonisation of Technical Requirements for Pharmaceuticals for Human Use (ICH), U.S. National Toxicology Program (NTP), World Health Organization (WHO), and IPCS. However, the detail of guidance from these organizations as it relates to design, execution, and analysis of immunotoxicity studies varies substantially. In addition to immunophenotyping, these guidance documents also discuss other hematology and clinical chemistry parameters, gross pathology, and more specific assays such as T-cell Dependent Antibody Response (TDAR), NK cell activity assays, macrophage/neutrophil function, and cell-mediated immunity.

Overall, there is minimal guidance for interpretation of immunophenotyping data among these technical documents. Generally, immunophenotyping data is used to inform overall immunotoxicity-related risk assessments, however, several guidance documents recommend integrating multiple streams of data rather than relying on a singular assessment. In fact, both the U.S. EPA and OECD, have presented a tiered approach instructing additional immunotoxicity assays be conducted following preliminary assessments and results. In contrast, when interpreting stand-alone immunophenotyping data for immunosuppression in the context of regulatory risk assessment, a weight of evidence (WoE) approach is recommended (WHO 2012). However, even among this guidance, interpretation of immunophenotyping data is limited and, at times, unclear. These guidance documents are detailed in Table 5.

The U.S. FDA has released an immunotoxicity testing guidance and framework, which is a modified version of ISO-10993, for the medical device industry along with FDA reviewers (FDA, 1999). In this guidance document, the FDA focuses primarily on hypersensitivity, chronic inflammation, immunosuppression, immunostimulation, and autoimmunity as “immunotoxic effects” (FDA, 1999). Further, the FDA defines an effect as immunotoxic or adverse if “it impairs humoral or cellular immunity needed by the host to defend itself against infectious or neoplastic disease (immunosuppression) or it causes unnecessary tissue damage (autoimmunity, hypersensitivity, or chronic inflammation)”, which is intended to incorporate the balance the immune system maintains with other body systems (FDA, 1999). Regarding immunophenotyping, as part of the guidance it can be used to assess immunotoxicity, specifically to reevaluate histopathology at the single cell level directed towards surface markers present on major immune cells populations (T-cells, B-cells, NK cells, macrophages) known to be involved in humoral and cellular immune responses (FDA, 1999). However, the FDA stated that “functional assays provide a more direct measure of immune system activity, and generally are more important than tests for soluble mediators, which are more important than phenotyping” (FDA, 1999). More recently, the FDA updated guidance for industry regarding nonclinical evaluation of the immunotoxic potential of pharmaceuticals, of which immunophenotyping is discussed (FDA, 2023). Specifically, the FDA stated that pharmaceutical drug impacts on the immune system, including immunosuppression and immunostimulation, can be evaluated by assessing the impact for immune cell subpopulations and that a WoE approach should be applied, further aligning with ICH S8 (described below).

The most comprehensive guidance for use and interpretation of immunophenotyping data was developed by IPCS. In their most recent Guidance for Immunotoxicity Risk Assessment for Chemicals, IPCS presented a framework for interpreting available human and laboratory animal data for the assessment of immunosuppression using a WoE approach (WHO 2012). Briefly, as outlined within the document, immunological data should be categorized and evaluated in order of most predictive value to least (WHO 2012: Fig. 4.1). Hematological evidence ranks fifth after epidemiological evidence, evidence of host resistance to infections or tumors, functional immunological evidence, general or observational immune assays, and followed only by histopathological evidence, and immune organ weight data. The evaluation of each category of evidence, including negative findings, provides the basis for the WoE and conclusions regarding the potential hazard. When it is concluded that an immunosuppression hazard exists, the most sensitive endpoint for a biologically plausible and significant response (critical effect) must be identified.

The IPCS guidance for interpreting hematology reports that only severe hematological changes alone are sufficient to demonstrate adverse immunosuppression and are appropriate for derivation of an effect level, but otherwise should not be used for that purpose. However, hematological changes 1) may provide MOA information to support a biologically plausible description of immunosuppression, or 2) may provide additional support for the WoE of immunosuppression if consistent with histopathological evidence (WHO 2012). Overall, IPCS states that the assessor should be less concerned whether values from the exposed population fall within typically broad historically normal ranges than whether the changes are statistically different from values obtained in an appropriately matched control population or whether there is a shift in the number of individuals who fall outside of the normal range when evaluating routine immune system data collected during epidemiological studies or routine toxicity testing.

In their preparation of toxicological profiles of various chemicals, ATSDR derives minimal risk levels (MRLs). Abadin et al. (2007) reported that of the 346 MRLs derived, only 15 (for 11 chemicals) were based on immunological effects. For immunological endpoints, ATSDR distinguishes morphological and functional changes. That is, cells that mature in the lymph nodes, spleen, and thymus “may or may not” be associated with functional changes in the immune response (Abadin et al., 2007).

The ICH report on immunotoxicity studies for pharmaceuticals offers guidance for results from standard toxicity studies such as hematological changes, alteration in immune system organ weights and/or histology, changes in serum globulins, increased incidence of infections, and increased occurrence of tumors that may be viewed as a sign of immunosuppression in the absence of other plausible causes. In addition, the use of flow cytometry is described for identification and/or enumeration of leukocyte subsets from lymphoid organs and peripheral blood. This guidance, updated in 2005, has been widely adopted and is now implemented in Europe, US, Canada, UK, Japan, and China.

According to guidance from NTP, functional effects should be weighed more heavily than observational parameters such as alterations in cell counts (*i.e.,* immunophenotyping). Per the NTP guidance, there are several categories or levels of evidence for immunologic hazard of chemicals that can be used to summarize study findings: “two categories for positive results (clear evidence and some evidence); one category for uncertain findings (equivocal evidence); one category for no observable effects (no evidence); and one category for experiments that cannot be evaluated because of major design or performance flaws (inadequate study)” (Germolec et al., 2022). Within the categories deemed positive results (clear evidence and some evidence), dose-related observations of functional changes are required. Observational, dose-related changes within a single parameter without functional effects are considered equivocal evidence of toxicity to the immune system. According to NTP guidance, it can be concluded that there is no evidence of toxicity to the immune system when studies of appropriate experimental design and conduct show no evidence of biologically relevant effects on the immune system related to the test article. In addition, NTP guidance specifies the consideration of other factors such as biological plausibility and insights from supportive studies (Germolec et al., 2022).

More recently in the scientific literature, Germolec et al. (2022) published a consensus of scientific evidence describing key characteristics of agents known to cause immunotoxicity in which applicable assays and techniques were described in the context of each characteristic. In this paper, the authors highlight the use and application of immunophenotyping for assessing these characteristics. Specifically, they highlight immunophenotyping when assessing immunosuppression and altered immune cell trafficking, when used in combination with other techniques that provide anatomic pathology end points (Germolec et al., 2022). The authors note that alteration of a key characteristic does not automatically equate to immunotoxic potential, and that a holistic and WoE approach should be employed by leveraging multiple techniques targeting multiple key characteristics. While Germolec et al. (2022) presents a novel viewpoint on immunotoxicological assessments in response to various exogenous hazards, there is minimal regulatory guidance related specifically to interpretation of immunophenotyping data.

As noted above, guidance for the interpretation of findings related to immunophenotyping data, particularly when small but statistically significant changes in one or more phenotypes occur, is often problematic, and it is unclear the level of change that might constitute an adverse health effect. Overall, guidance documents reference the use of non-functional assays, such as immunophenotyping, be used to inform overall immunotoxicity assessments along with functional assays. However, guidance as to how immunophenotyping results can guide or inform downstream immunotoxicity assessments is unclear or missing. Given that immunophenotyping is one of the most commonly used tests to evaluate immunological changes in human studies, there is greater need for clarity and guidance when applying these data toward risk assessments.

## Case studies

Case studies were selected with the intent of highlighting some of the issues and opportunities related to immunophenotyping including data interpretation and importance for using a WoE approach to facilitate proper use in risk informed decision making. The following case studies are based on studies conducted in experimental animals or *in vitro* test systems following exposure to industrial chemicals and therapeutic where the observations reported include:

a) decrease in certain immunophenotype cells with no clear direct physiological consequence
b) lack of sensitivity for immunophenotyping in detecting immunotoxicity
c) similar effects observed among studies for certain immune cell types with no clear understanding of the MOA
d) interpretation of functional immune responses is improved through concurrent quantification of immune cell populations

### Asphalt, sulfonated, sodium salt

Asphalt, Sulfonated, Sodium Salt (SAS) is a drilling mud additive consisting of a diverse distribution of sulfonated alkyl aryl hydrocarbon constituents with a molecular weight range between 500–3,000 Da. A 90-day sub-chronic toxicity study was conducted for SAS at doses of 100, 300, and 1,000 mg/kg/day in rats exposed orally (Charles River Laboratories, 2021). The findings of this study are reported along with considerations explaining the difficulty interpretating effects associated with a marginal, but statistically significant, reduction in eosinophil levels based on peripheral blood hematological complete blood counts and whether this constitutes an adverse effect for NOAEL determination in the absence of any other toxicological findings. The question of a small decrease in eosinophils is important since these cells are particularly important in protecting against helminth infections and play a major role in allergic diseases.

Authors of the study (Charles River Laboratories, 2021) reported a statistically significant (p ≤ 0.05) decrease in eosinophils at all dose groups only in male Wistar Han rats (Table 6). Eosinophil counts for male and female rats were slightly lower than controls in the recovery group but were not statistically significant. When the eosinophil counts in males were compared with the historical controls, the values were within the normal range. This is informative when assessing the variability relative to a particular endpoint, especially when precedent is typically given to concurrent controls. In this instance, eosinophil counts in all male dose groups were within historical control ranges, and it is possible that the reported statistical significance in males could be explained by a high control group mean and complicated by the coefficient of variation due to the small numbers of eosinophils relative to other cell types (Dale, 2023). There were no corroborative histopathological findings, and an identified reproductive and developmental screening toxicity study in rats reported no hematological effects at similar doses suggesting a lack of consistency. Taken together, the inherent variability for a particular endpoint and assay, the lack of corroborative histopathology and lack of consistency in a reported effect (no significant decrement in females vs. males, and no decrease in males in a second repeat dose study) indicate the observed decrease in eosinophil counts in male rats dosed with SAS is more likely a chance finding rather than an adverse effect.

**TABLE 6:** Mean Eosinophil counts (10^9^/L)^a^ from male and female rats in a 90-day oral gavage study of Asphalt, Sulfonated, Sodium Salt with recovery.

Dose levels (mg/kg/day)	Dose group		Recovery group	
	Female (n = 10)	Male (n = 10)	Female (n = 5)	Male (n = 5)
Control (Elixir water)	0.095 ± 0.099	0.125 ± 0.050	0.072 ± 0.050	0.070 ± 0.007
100	0.056 ± 0.022	0.080 ± 0.025*	—	—
300	0.054 ± 0.021	0.071 ± 0.026**	—	—
1,000^	0.050 ± 0.019	0.066 ± 0.029**	0.040 ± 0.007	0.056 ± 0.013

ANOVA and Dunnett: * = *p* ≤ 0.05; ** = *p* ≤ 0.01.

^n = 9 for males at 1,000 mg/kg/day.

^a^ Historical control range for Wistar Han rats (period 2018–2021): Male (N = 227): Mean = 0.085, 5th – 95th percentile 0.030–0.170, range (0.02–0.22); Female (N = 226): Mean = 0.062; 5th – 95th percentile 0.020–0.141; range (0.02–0.33); Historical control data provided by Charles River Laboratories, Inc. (Den Bosch B.V.)

**TABLE 7:** Treatment related effects associated with sulfolane on the immune system from multiple studies conducted.

Study	Dose levels (mg/kg/day)	Species and route	Reported NOELs (mg/kg/day)	Effect
Huntington Life Sciences, 2001	2.1, 8.8, 35.0 131.7 (Males) 2.9, 10.6, 42.0, 191.1 (Females)	Rat, dietary oral	2.9 (Females)	None in males Decreased total and differential WBC counts (lymphocytes, basophiles, monocytes, and LUCs) in females
Ministry of Health and Welfare Japan, 1996	60, 200, 700	Rat, oral	200 (NOAEL – Females)	Decreased spleen weight in females
Watson et al. (2021)	30, 100, 300, 1,000	Rat, dietary oral	30 (Females)	Reduced NK cell activity
Labcorp (2023)	80, 200, 500	Rat, oral	200 (Females)	Decreased NK cells in males and females

### Benzo(a)pyrene-phenanthrene

Benzo(*a*)pyrene [B (*a*) P] and phenanthrene are structurally related polycyclic aromatic compounds (PACs), produced via both natural and anthropogenic sources from incomplete combustion emissions of organic materials. While phenanthrene, unlike the human carcinogen B (*a*) P, is not classified by IARC (2010); IARC, (2016) as a human carcinogen, it contains a bay region area like many carcinogenic PAHs as well as demonstrates similarities in metabolism and detoxification, including P450 induction, (Hecht, 2002; Nota et al., 2009). In laboratory animal studies, B (*a*) P is associated with immunotoxicity, particularly suppression of humoral immunity while phenanthrene was shown to not be immunotoxic (Dean et al., 1983; Temple et al., 1993; Silkworth et al., 1995).

As mentioned previously, one concern in using immunophenotyping studies in identifying immunotoxic chemicals is a perceived lack of sensitivity. This was exemplified in studies conducted by NTP where the immunotoxicity of B (*a*) P and phenanthrene were compared using identical experimental protocols (Johnson et al., 2017).

In 28-day studies in female B6C3F1/N mice, the benchmark dose lower limit (BMD_L_) for inhibition of the T-dependent antibody response (TDAR) to sheep red blood cells was approximately 15 times lower for B (*a*) P compared to phenanthrene using the same study designs (Rider et al., 2023). While immunophenotyping studies showed significant decreases in most cell types (panel 1 for T-cells, CD4 T-cells, CD8 T-cells, panel 2 for T-cells, B-cells, NK cells, and panel 3 for monocyte/macrophage, eosinophil, neutrophil) in mice following treatment with B (*a*) P at dose levels as low as 5 mg/kg/day, phenanthrene treated mice failed to show any significant phenotypic changes at dose levels up to 400 mg/kg/day, the highest dose level tested. The BMD_L_ for immunophenotyping changes following B (*a*) P treatment were >3 times higher than observed for the TDAR depending on the immune cell population examined, revealing sensitivity difference between the two immune tests (Johnson et al., unpublished data). In this case, the functional testing and immunophenotyping show that B (*a*) P is the more potent immunotoxicant. However, phenanthrene is also active as an immunotoxicant as evidenced by suppression of the TDAR in mice exposed to ≥50 mg/kg/day (Johnson et al., 2017), a finding that would not have been identified using immunophenotyping alone as phenotypic changes were not observed up to the highest dose tested of 400 mg/kg/day.

### Sulfolane

Sulfolane is a highly polar and stable organosulfur compound that is used as an industrial solvent for various applications. Applications include BTX (benzene, toluene, and xylene) extraction of aromatic hydrocarbons from refinery reformate, vapor suppressant in combination with hydrofluoric acid (HF), jet printing formulation, synthesis of pharmaceutical intermediates, and lithium batteries.

Several studies conducted with sulfolane suggest that leukopenia may be the most common and sensitive effect on the immune system based upon hematological or immunophenotyping, using flow cytometry (Total T-cells, CD4 T-cells, CD8 T-cells, B-cells and NK cells), to analyze various cell type populations. Huntingdon Life Sciences (2001) reported a mild to moderate dose dependent decrease in total and differential WBC counts (*i.e.,* lymphocytes, basophils, monocytes, and large unstained cells) in female rats during a 90-day drinking water study. Only spleen weights at the highest dose were significantly decreased and similar effects were not observed in males (Huntingdon Life Sciences, 2001). Watson et al. (2021) reported the most sensitive immune system related effect was a reduction in NK cell activity, up to 47%, in female F1 rats at doses greater than 100 mg/kg/day sulfolane in drinking water. A recent Extended One Generation Reproductive Toxicity Study found that the most pronounced hematological effect was a decrease in NK cells in F1 male and female rats dosed by oral gavage at 0, 80, 200, and 500 mg/kg/day. Spleen weights at 500 mg/kg/day were also decreased; however, there were no histological correlates (Labcorp, 2023). Upon closer evaluation of the spleen, almost all cell types demonstrated a shallow decreased dose response trend in total WBCs and leukocytes in absolute values across the immunophenotypes measured. The effects even at the highest dose reached a maximum of only (33%) of controls, which suggests this dose produced mild leukopenia. There are additional studies showing that exposure to sulfolane targets the immune system and may be associated with leukopenia (Anderson et al., 1977; OECD, 2004; Zhu et al., 1987).

There is currently no MOA determined for sulfolane that would account for the observed effects on the immune system. Despite lack of specific mechanistic data, it is plausible the observations regarding mild leukopenia reflect a general effect of solvents on the immune cell counts. For example, exposure to solvents like BTX have been associated with hematological changes that are described as chemical specific mechanisms (Cakmak et al., 2020; Aksoy, 1989). Although these mechanisms have yet to be fully elucidated, they are likely to be complicated by various pathways such as metabolism, growth factor regulation, oxidative stress, DNA damage, cell cycle regulation, and programmed cell death. Thus, while detailed mechanisms for sulfolane are not known, the mild leukopenia effect is biologically plausible bridging from other solvents. However, since the relative changes in NK cell levels are small and the adverse effect uncertain, there is reluctance to use the data as the primary POD basis for establishing an HBEL. Rather HBELs for sulfolane have been derived from other immune system effects at higher doses from the body of studies (TERA, 2014).

### Dexamethasone

Development and utilization of *in vitro* test systems for immunotoxicity assessment are rapidly expanding and offer opportunities to change the trajectory of safety assessment. Advantages offered by *in vitro* technologies include but are not limited to reduction in the use of animals in toxicity testing and elimination of interspecies extrapolation and uncertainty by using human cells. Recently a human whole blood immunotoxicity testing battery was developed to assess functional immune responses of innate and adaptive immune cells (Johnson, 2023). The test system was designed to assess NK cell and T-cell function in response to stimulation with viral antigens with the goal of determining impacts of toxins on these responses. It was essential to incorporate immunophenotyping (T-cells, CD4 T-cells, CD8 T-cells, NKT cells, B-cells, NK cells, monocytes, neutrophils and eosinophils using a single 8-color antibody panel) as part of the test system so that changes in population numbers could be used to interpret the functional changes. Treatment of the *in vitro* test system with dexamethasone was shown to cause a concentration dependent decrease in NK cell killing activity, T-cell activation in response to viral peptide stimulation, and viral peptide stimulated proinflammatory cytokine production. Flow cytometry was used to quantify immune cells and showed that the number of NK cell and T-cells were not affected by treatment with dexamethasone, solidifying the conclusion that deficits in immune function were responsible for the immunosuppression caused by dexamethasone (Johnson, 2023). Without the immunophenotyping, the question would remain if cytotoxicity, proliferation, and/or functional changes were responsible for the effects of the toxin.

### Proposed decision flow

**FIGURE 2:**
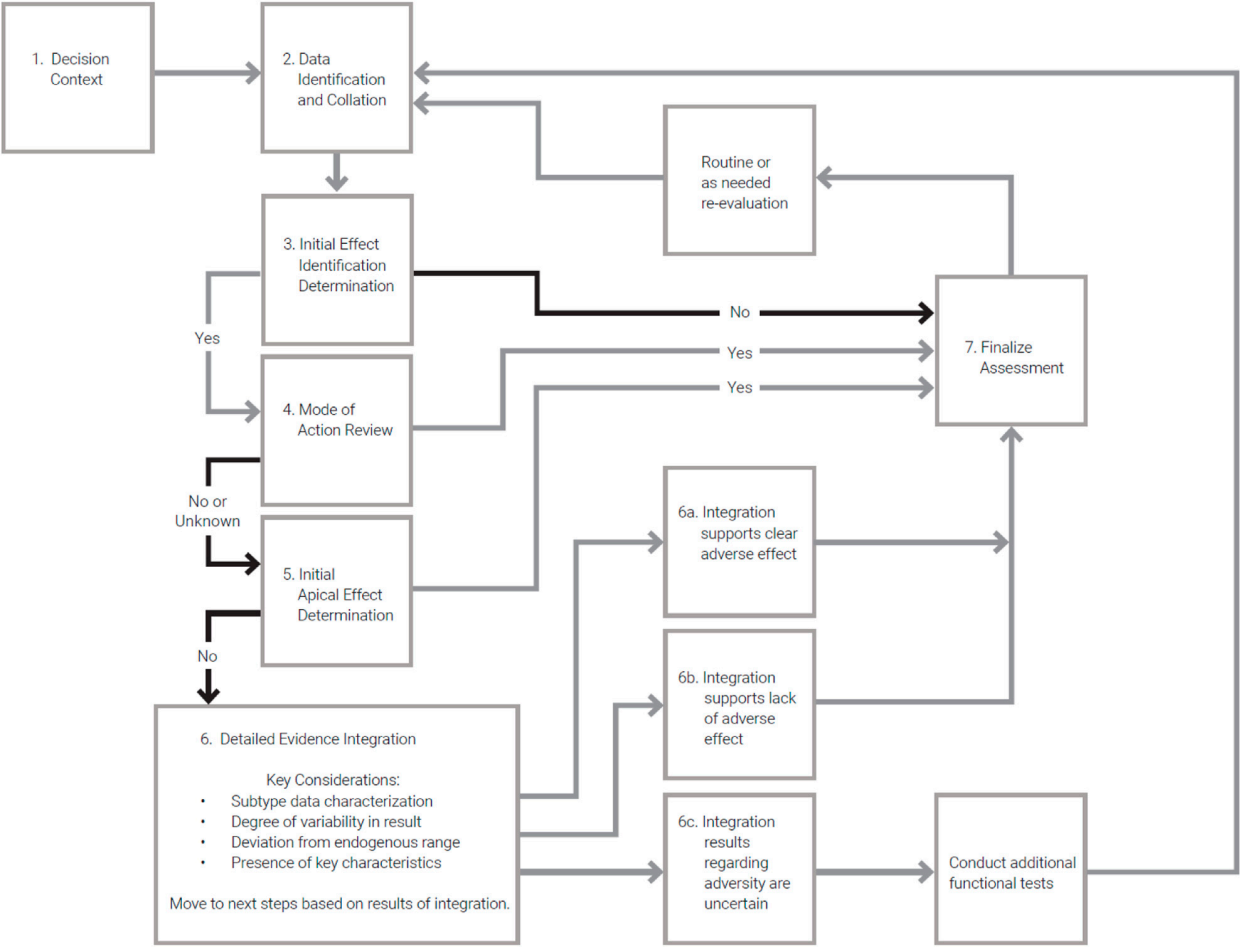
Flow diagram illustrating the decision logic for immunophenotyping data assessment described in Table 7.

Existing guidelines related to assessment of immunophenotyping endpoints (Table 5) provide high-level descriptions of the conditions under which the end point should be reviewed, potential testing methods and protocols to be employed, and general considerations for evaluation of results. We propose a decision logic that provides a workflow to augment these other guidelines (Table 8). The decision flow is aimed at providing the sequence of steps one might pursue in making decisions related to risk informed decision making for immunosuppressive effects based on immunophenotyping data, including adverse effect level determination and identification of data gaps that support additional more determinative testing (Figure 2).

**TABLE 8:** Decision logic for immunophenotyping data assessment.

Step	Considerations
1. Decision Context	Risk assessment decisions require contextualization. This is typically part of problem formulation in current risk assessments. The problem formulation defines the question one is trying to address and identifies relevant guidelines and data streams to be pursued. The outcome is a clear description of the decision context for the data evaluation
2. Data Identification and Collation	Developing and implementing a strategy for data identification, review, and integration is a foundation of systematic review. This step reflects development of the strategy for ensuring all relevant data are identified and evaluated according to specified criteria related to relevance and reliability. The outcome is a culled data set reflecting the information to be used in the immunotoxicity assessment
3. Initial Effect Identification Determination	When data are arrayed an initial screening data review is often valuable to efficiently identify the need for immediate follow up questions or to set aside further investigations. This initial review would first determine if any significant changes regarding immunophenotype were observed. This will often include a statistical and biological perspective. In general, this screening review is aimed at verifying no observed relevant effect. If that cannot be affirmed in the data with confidence, then additional evaluation is performed
4. Mode of Action Review	The observed immunophenotype changes should be made through the lens of the MOA if it is known or hypothesized. MOA knowledge and linkage to clear AOPs is helpful in determining whether the observed effect is anticipated and can be evaluated within an established AOP that incorporates general perspective from the field on adversity and clinical relevance of a finding
5. Initial Apical Effect Determination	Even in the absence of more nuanced understanding of biology via an AOP, in some cases the nature of the observed change is sufficiently clear as to identify an adverse effect condition. Example would include large changes in WBC count or sub-type counts that have already been demonstrated in the general pharmacology and toxicology literature to be clinically relevant degrees (or patterns) of change for function. This determination leverages the current precented on defining “adverse” effects for risk assessment
6. Detailed Evidence Integration	In many cases, the MOA of a chemical is not known and a clear determination of adverse or non-adverse changes is not possible (*i.e.*, for small magnitude of changes or complex WBC subtype changes). A detailed evidence integration is required Four common lines of evidence applied in such decisions are shown and application of evidence integration is described in the case studies in this manuscript
7. Disposition of Findings	The results of the detailed evidence integration will often lead to one of three determinations as shown in Figure 2
8. Re-evaluation of Assessment	Similar to other safety and risk assessments, re-evaluation should be performed on a routine basis or as needed if new data were generated, or assessment methodology has changed

The decision logic and flow chart (Table 8; Figure 2) presents a step-by-step process to assess the importance and quality of relevant immunophenotyping data in relation to safety or risk assessments. Further, the developed logic aids the user in understanding the necessary steps to utilize these data and how to best incorporate findings into the overall assessment. While this logic does not attempt to answer all questions related to the use of immunophenotyping data, it presents clear next steps for incorporation into immunotoxicity assessments and was applied in the case studies described above. As such, this logic attempts to fill a gap currently present in regulatory guidance.

## Conclusion

Immunophenotyping adds value to immunotoxicity studies as it increases overall screening predictability when combined with other immune tests and can help identify the MOA. However, because of the large inherent variability in the use of immunophenotyping studies for this purpose, there is an elevated risk of inaccurate interpretation of small but statistically significant changes in immune cell populations. The examination of clinical relevance of changes from a pathophysiology perspective should be considered as it provides the strongest opportunity for evidence integration. In many cases, an MOA analysis approach linked to an AOP is ideal. The identification of early key events and linkage to the adverse biology can be examined using approaches derived from the modified Bradford Hill considerations. Consultation with an experienced immunologist or toxicologist with specific experience in immune system evaluation is typically required to support such judgements. The level of confidence in developing an adversity decision is based on the degree of change, the pattern of subtypes affected and evidence from supporting studies. The resulting integration of evidence is an important step in developing a final safety assessment related to immunological effects.
